# Peripheral Circumferential Retinal Detachment after Pars Plana Vitrectomy: Complications and Management

**DOI:** 10.3390/jcm11164856

**Published:** 2022-08-18

**Authors:** Cherng-Ru Hsu, Chung-May Yang

**Affiliations:** 1 Department of Ophthalmology, National Taiwan University Hospital, Taipei 100, Taiwan; 2 Department of Ophthalmology, Tri-Service General Hospital, National Defense Medical Center, Taipei 114, Taiwan; 3 Department of Ophthalmology, National Taiwan University College of Medicine, Taipei 100, Taiwan

**Keywords:** anterior proliferative vitreoretinopathy, corneal opacity, hypotony, peripheral circumferential detachment, rubeosis iridis

## Abstract

Purpose: This study aimed to evaluate treatment outcomes and complications of peripheral circumferential retinal detachment (PCD) after successful vitrectomy. Methods: Eyes diagnosed with PCD after pars plana vitrectomy (PPV) were retrospectively reviewed. The patient demographic data, complications, management, and treatment outcomes were collected and analyzed. Results: The mean follow-up duration was 18.0 ± 11.9 months. BCVA ranged from light perception to 0.1 (median: counting fingers at 40 cm). Major complications included rubeosis iridis (seven eyes), vitreous hemorrhage (five eyes), hyphema (five eyes), corneal decompensation (three eyes), hypotony (two eyes), and neovascular glaucoma (two eyes). All eyes underwent peripheral retinectomy to remove the detached retina and release traction. Complete retinal reattachment was achieved in all eyes. The final BCVA ranged from hand motion to 0.1 (median: counting fingers at 30 cm). Conclusion: PCD may be associated with delayed-onset complications, causing severe loss of vision. Proper management, including peripheral retinectomy, may preserve visual function.

## 1. Introduction

Pars plana vitrectomy (PPV), with or without combined scleral buckle (SB), has been widely used to treat complicated rhegmatogenous retinal detachment (RRD). Many clinical variables, including choroidal detachment, significant hypotony, grade C proliferative vitreoretinopathy (PVR), four detached quadrants, and large or giant retinal breaks, have been identified as risk factors for surgical failure [1].

Recurrent retinal detachment (RD) usually occurs within 3 months after primary surgery [2]; recurrent RD that occurs 6 or more weeks postoperatively has been defined as late-onset recurrent RD [3]. Previous studies found that vitreous base traction was a major cause of recurrent RD [4]. With recent advancements in surgical techniques and tools, such as the use of small-gauge instruments and bimanual maneuvers to release tractional forces, the surgical outcome of post-vitrectomy retinal detachment has been enhanced; however, RD recurrence remains an important issue [5]. Among various patterns of recurrent RD, there is a specific form in which the RD is present circumferentially in the peripheral area. This peripheral circumferential detachment (PCD) is defined and characterized by its limitation to the periphery in a circumferential fashion. This unique configuration is generally attributed to the equatorial laser-induced chorioretinal scars obtained in previous surgeries, which prevent the RD from extending posteriorly [6]. PCD may initially be involved in the inferior periphery and may later extend circumferentially to form a tube-like configuration. In a manner distinct from classic re-detachment, which promptly or gradually advances toward the posterior retina or involves the macula, central vision remains unaffected due to the attachment of the posterior pole; however, specific late complications may occur, resulting in loss of late visual acuity (VA) [7].

In the present study, we evaluated the clinical characteristics and complications associated with PCD after successful vitrectomy and investigated the anatomical and functional treatment outcomes.

## 2. Materials and Methods

This retrospective case series study was conducted by reviewing the medical records of consecutive patients treated at National Taiwan University Hospital between January 2000 and December 2020. The study adhered to the tenets of the Declaration of Helsinki and was approved by the institutional review board of the National Taiwan University Hospital. The requirement for informed consent was waived.

We defined PCD as a form of recurrent RD limited to the periphery in a circumferential or tube-like fashion by old equatorial laser-induced chorioretinal scars. PCD was diagnosed mainly based on the findings of indirect ophthalmoscopy, B-scan ultrasonography, or optical coherence tomography (OCT). A standard three-port 23-gauge or 25-gauge PPV was performed for each patient by one senior surgeon (C.-M.Y.). The inclusion criteria were as follows: (1) recurrent RD after vitrectomy showing the distinct features of PCD and (2) development of late complications causing VA change. Patients were excluded if they had a follow-up duration of less than 6 months. Demographic data, including age, sex, number of previous surgeries, previous surgical procedures, onset time of symptoms, operation findings, complications, and follow-up duration, were recorded. Comprehensive ophthalmic examinations, including best-corrected visual acuity (BCVA) measurements, intraocular pressure (IOP) measurements, slit-lamp biomicroscopy, indirect ophthalmoscopy, and dilated color fundus photography (CR-DGi Image Viewer; Canon Inc., Tokyo, Japan), were performed. The outcome measurements were the anatomical outcome, indicated by whether the retina remained re-attached or not at the last visit, and the functional outcome, represented by the best-corrected visual acuity at the last visit. Successful re-attachment of PCD was defined as the attachment of the retina with or without endotamponade agents for at least 6 months of follow-up.

Statistical analyses were conducted using SPSS statistical software version 26 (SPSS Inc., IBM Company, Chicago, IL, USA). The mean and standard deviation were calculated for continuous variables. Categorical variables are expressed as frequencies and proportions. To quantify visual acuity in eyes with low vision, counting fingers’ visual acuity was assigned the logMAR units of 1.7, 1.85, 1.90, and 2.00, based on the different distances of measurement and hand motion visual acuity of 2.30 [8].

## 3. Results

Nine eyes in nine patients (six men and three women) were included in the analysis. Patient ages ranged from 31 to 74 years (mean, 52.3 ± 13.9 years). The average follow-up duration was 19.6 ± 12.5 months (range: 6–48 months; median: 15 months). The mean number of previous operations was 2.3 ± 0.47 (range: 2–3). The average onset time of symptoms ranged from 3 to 96 weeks (mean, 130.4 ± 155.7 weeks). The preoperative BCVA was light perception in three eyes, hand motion in one eye, counting fingers in two eyes, and >0.01 in three eyes. The mean preoperative IOP was 17.1 ± 15.3 mmHg, including three eyes (22.2%) with an IOP ≤ 5 mmHg and two eyes (22.2%) with an IOP > 30 mmHg (Table 1).

**Figure 1:**
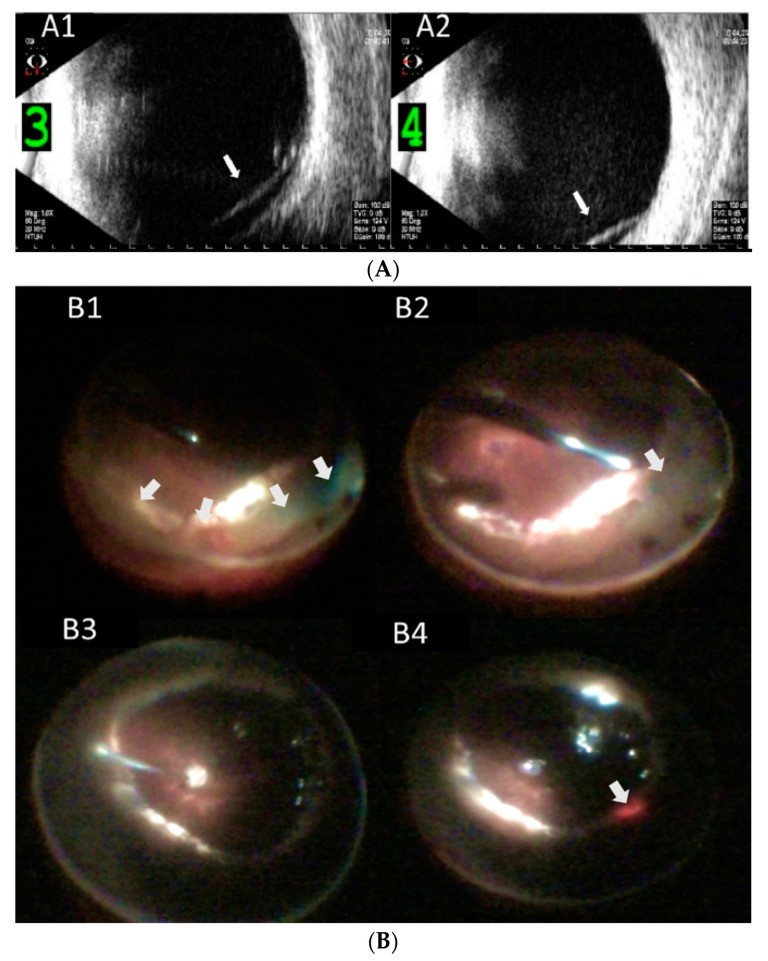

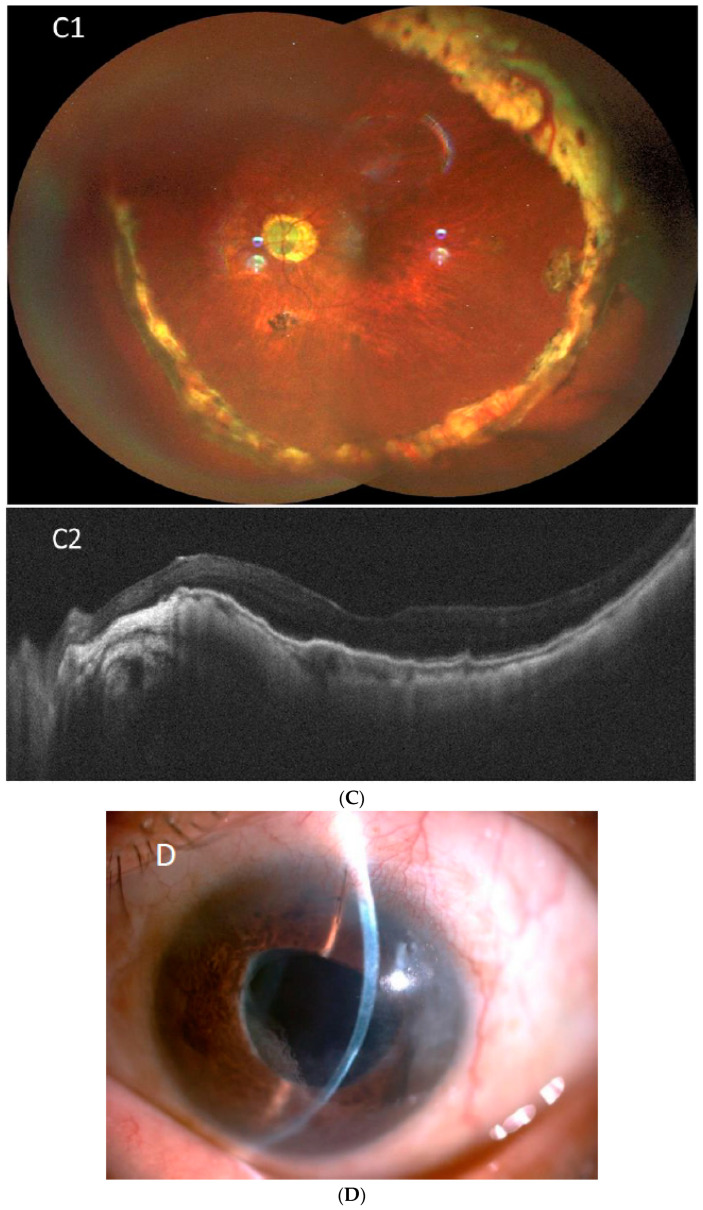
Representative imagings of peripheral circumferential retinal detachment (RD) in case 6. (**A**) Preoperative B-scan ultrasonography shows RD in inferior (**A1**) and (**A2**) nasal periphery (arrows); (**B**) intraoperative pictures show (**B1**) 270 degrees of peripheral RD (arrows), (**B2**) reti-nectomy of peripheral retina (arrow), (**B3**) fluid-air exchange, and (**B4**) supplementary laser pho-tocoagulation (arrow); (**C**) postoperative wide angle fundus photography shows attached retina under silicone oil (**C1**), and (**C2**) optical coherence tomography imaging shows the macula remained attached; (**D**) external eye photography shows superficial corneal scar from endothelial cell loss as a late-onset complication.

The major complications included rubeosis iridis in seven eyes (77.8%), vitreous hemorrhage in five eyes (55.6%), hyphema in five eyes (55.6%), corneal decompensation in three eyes (33.3%), hypotony in two eyes (22.2%), and neovascular glaucoma in two eyes (22.2%). During surgical interventions for PCD, peripheral RD > 180° was noted in eight eyes (88.9%). Anterior PVR with retinal-ciliary adhesions was observed in three eyes (33.3%). Peripheral retinectomy to release the tractional tissue was performed in all nine eyes. Silicone oil (SO) was used for endotamponade in eight eyes (88.9%), and perfluoropropane (C3F8) was used as a gas tamponade in the remaining one eye (11.1%). Combined intravitreal anti-vascular endothelial growth factor (anti-VEGF) injection was performed in four eyes (44.4%), with either rubeosis iridis or hyphema with vitreous hemorrhage. After surgery, the retina was completely reattached with the regression of rubeosis iridis and resolution of neovascular glaucoma. One eye (case 6) had developed recurrent vitreous hemorrhage 30 months after the operation for PCD. Intravitreal injection of anti-VEGF and triamcinolone was prescribed initially, and one week later, and the vitreous hemorrhage had gradually resolved. The final BCVA at the last follow-up was hand motion in two eyes, counting fingers in three eyes, and ≥0.05 in four eyes. The mean final IOP was 5.7 ± 1.7 mmHg, including two eyes (22.2%) with IOP ≤ 5 mmHg and no eyes with IOP > 30 mmHg. Corneal decompensation was observed in three eyes during follow-up; among them, one eye (case 8) received corneal transplantation (Descemet stripping automated endothelial keratoplasty (DSAEK)) for severe corneal opacity (Table 1 and Table 2). Corneal decompensation was related to recurrent hyphema and hypotony after multiple vitrectomies with SO tamponade in one patient (case 5), long-term SO-corneal touch before the development of PCD in one patient (case 6), and AC-IOL implantation and uveitis-glaucoma-hyphema syndrome in one patient (case 8). An example of PCD (case 6) is shown in Figure 1.

## 4. Discussion

In this study, the complications and treatment results of a unique pattern of recurrent RD after vitrectomy for RRD were reported. Recurrent postoperative RD was characterized by peripheral circumferential RD with a partial or complete tube-like configuration. Vision-affecting complications are usually late onset, including rubeosis iridis, vitreous hemorrhage, hyphema, corneal decompensation, hypotony, and neovascular glaucoma. The final vision was guarded in the majority of cases due to both anterior and posterior segment abnormalities despite careful removal of the peripheral detached retinal tissue, with or without SO tamponade.

A previous study observed that 10–13% of patients required additional interventions for recurrent RD despite initial anatomical success after a single operation [9,10]. For multiple recurrences of RD, Enders et al. found that patients with initial recurrent detachment were exposed to a 21–26% risk of recurrent RD after each additional surgical procedure aimed at RD repair [11]. Multiple surgical procedures increase intraocular inflammation and exacerbate the risk of PVR formation [12]. Preoperative anterior PVR and more severe PVR grades were associated with a higher incidence of RRD recurrence and worse visual outcomes [13,14]. In our series, all eyes underwent more than one vitreoretinal surgery for retinal attachment. Although PVR is usually associated with higher severity and macula involvement, RD is sometimes limited to the peripheral retina, as PVR is mainly anterior in its extent, and the peripheral circumferential heavy laser or SO in the vitreous cavity prevents the RD from advancing into the posterior retina. In our study, all nine eyes received supplemental laser retinopexy in the previous untreated peripheral retina during intraoperative management of PCD to achieve 360-degree laser retinopexy. As shown in a previous study [15], 360 degrees of laser retinopexy reduced the incidence of retinal detachment after silicone oil removal in vitrectomized eyes for rhegmatogenous retinal detachment. Although we did not remove all of the silicone oil in most SO-filled eyes during the last visit in our study, 360 degrees of laser retinopexy could prevent unseen breaks or avoid the formation of new breaks.

Unlike most major recurrent RDs, central vision remains initially unaffected in PCD due to the attachment of the macula. However, PCD may cause late-onset complications. In this study, the main complications associated with late-onset PCD included rubeosis iridis, intraocular hemorrhage, chronic hypotony, and neovascular glaucoma. Rubeosis iridis in PCD may result from chronic ischemia and inflammation. Posterior segment ischemia and increased angiogenic factors resulting from RD are attributed to rubeosis iridis, which has been reported to occur as early as 3 weeks after PPV [7]. Ischemia may also induce NV in the peripheral retina. Chronic inflammation after multiple surgeries enhances the secretion and interaction of cytokines and angiogenic factors, which may contribute to and further worsen rubeosis iridis. A previous study reported the possibility of undetected peripheral retinal detachment or anterior PVR in the development of rubeosis iridis in eyes without retinal vascular disease [7]. Another possible factor leading to rubeosis may be the direct extension of fibrovascular tissue from the anterior PVR. The events that occur after rubeosis include obstruction of the trabecular meshwork and hyphema. In our cohort, 77.8% (7/9) of the patients had rubeosis iridis, and 55.6% (5/9) had hyphema. However, only two patients had glaucoma, while chronic hypotony occurred in two eyes in our series. Hypotony has been described in multiple operations, inflammation, and ciliary detachment, which are associated with inflow disturbance and outflow enhancement in RD [16]. The intraocular pressure depends on the balance between outflow obstruction and inflow production. Because NV and rubeosis take time to develop into PCD, the initial visual acuity may not decrease. Patients may seek help after sudden onset of vision loss due to intraocular hemorrhage or intraocular pressure change. The onset of complications varied among the study patients. The time of onset of PCD beyond one year was found in three patients (cases 6, 7, and 8) with poor baseline VA. In addition, vitreous hemorrhage with hyphema was noted in these three patients. It is possible that peripheral detachment occurred earlier than was noticed by major vision changes caused by hemorrhage in the eyes. The high complication rate may be due to the low patient numbers, as we only included patients with declined VA in our study. Eyes with a mild form of PCD that had a lesser extent of circumferential detachment of less than 150 degrees may not cause VA change and thus were not included. Therefore, the incidence of PCD may be underestimated. The more extensive the circumferential detachment, the higher the complication rate.

To prevent PCD, it is essential in the primary operation to release peripheral traction, trim, and support the vitreous base and properly seal the peripheral breaks. Once PCD complications occur, retinectomy of the detached and ischemic retina should be performed to eliminate neovascularization stimuli and release retino-ciliary traction. Intravitreal anti-VEGF injections may be administered at the end of surgery. Furthermore, SO injection to tamponade the retina may decrease hypotony-induced macular striae formation and prevent angiogenic factors from reaching the anterior segment. Scleral buckles may not be needed, as extended retinectomy alleviated the retinal-ciliary traction in our series. The combined PPV and encircling band reduced the traction within the vitreous base; however, this may cause anterior segment ischemia that aggravates the PCD-related complications, such as rubeosis iridis, vitreous hemorrhage, or hyphema.

After surgery, all nine eyes achieved retinal reattachment following extended retinectomy with SO-filled or C3F8 endotamponade at the end of the surgery. Intraocular silicone oil has previously been established as a long-term tamponade agent in treating severe vitreoretinal diseases as a last-resort option in selected patients. The recurrent vitreous hemorrhage observed in case 6 may reflect the unstable condition despite the successful re-attachment. Hence, SO tamponade may offer a more stable condition in these complicated cases. In our series, intraocular pressure was within the normal range in seven eyes, while two had hypotony at the last follow-up visit. Hypotony, which occurred in cases 2 and 5 despite an SO-filled vitreous cavity, may have been related to the 360° retinectomy that was performed and the annular anterior PVR before the operation. Decreased aqueous humor production secondary to ciliochoroidal effusion or ciliary body dysfunction secondary to anterior PVR with an increased posterior outflow of the subretinal fluid from retinectomy may account for this hypotony [17]. We reported four cases of PCD in our previous study [6]. Despite the lately improvement of modern surgical instruments, PCD developed with similar characteristics. In addition to a larger number of cases and a longer complication-onset time compared to our previous study, a significant feature of the present study was the development of corneal decompensation in one-third of the cases. Corneal abnormality is a complication of vitrectomy with gas or silicone oil tamponade [18]. Previous research has found that the development of corneal edema and keratopathy due to endothelial dysfunction is a consequence of insufficient nourishment from reduced aqueous humor circulation [19]. However, Banaee et al. did not find keratopathy to be more frequent in hypotonic eyes or in those with retained silicone oil. Instead, baseline corneal condition, multiple vitrectomies, and trauma history were significantly associated with the development of keratopathy [20]. Similarly, corneal opacity was noted in three patients in our series, and one of them (case 8) underwent DSAEK. AC IOL implantation, as in this patient, and older age may aggravate corneal decompensation. In addition, persistent hyphema after vitrectomy with corneal blood clot staining was observed in the three patients with corneal opacity.

Visual acuity improved in case 6 despite mild corneal opacity and in case 8 after corneal transplantation. Case 5 had corneal opacity and worse final visual acuity postoperatively despite reattachment of the retina. In contrast to cases 1–4, both cases 5 and 9 showed no improvement in VA after the operation. The predictability of functional outcomes remains poor because of the wide range in interindividual postoperative visual acuity. The relatively longer onset time found in cases 6–8 may have been due to several factors. The poor initial VA in these three patients might have delayed the awareness of vision change. In addition, it might take longer for the limited detachment area to generate sufficient angiogenic factors required for the development of neovascularization in the posterior and anterior segment structures. These possible reasons may explain the coexistence of hyphema and vitreous hemorrhage in cases 6–8. Although visual acuity of ultimate success in these extreme cases was limited, long-term follow-up demonstrated stable anatomical outcomes.

There were several limitations in this study. First, selection bias may exist due to the retrospective nature and monocentric setting of this study. Second, due to the rarity of PCD, despite the twenty-year interval, only a limited number of eyes were enrolled in our study. Furthermore, the follow-up time of individual patients was highly variable and was driven by subjective need rather than a regular follow-up time.

In conclusion, the results of this study suggest that prompt recognition and timely treatment of such late-onset complications in PCD are important to preserve a certain degree of visual function. However, functional outcomes in patients affected by PCD are very poor. Careful removal of the traction force in the vitreous base with retinectomy to release contractile tissue assists in restoring the retinal architecture and lowers the risk of persistent NV stimuli from the devitalized membrane.

## Figures and Tables

**Table 1:** Clinical characteristics of cases of peripheral circumferential retinal detachment.

No.	Age	Sex	R/L	Pre Op VA (LogMAR)	Pre Op IOP	Hyphema	NVI	VH	Previous OP Procedures	Lens	Onset Time (Week)	OP Procedures	Findings	Ant. Adhesion	F/U (M)	Final VA (LogMAR)	Final IOP	Notes
1	39	M	R	LP(+)	9	+	+	+	PPV*2	Dense cataract	14	PPL + 360° retinectomy + SO	360° pRD	-	9	HM (2.3)	WNL	
2	60	M	R	CF/40 cm (1.85)	1	-	+	-	SB*1, PPV*1, Lens extraction	PC-IOL	12	360° retinectomy + SO	360° pRD	+	12	0.1 (1.0)	5	
3	46	M	R	0.05 (1.3)	39	-	+	-	PPV*2, SB + SO + PPL	Aphakia	26	270° retinectomy + SO refill	225° pRD	+	6	0.1 (1.0)	WNL	
4	40	M	R	0.025 (1.6)	WNL	-	+	-	PPV*1, SB + SO + PPL	Aphakia	22	180° retinectomy + PI + SO refill	180° pRD	-	22	0.05 (1.3)	WNL	
5	71	F	R	CF/100 cm (1.7)	6	+	+	+	PPV*3, SO, Lens extraction	PC-IOL	20	360° retinectomy + SO + IVIA	360° pRD	+	15	HM (2.3)	4	IVIA*3, CO
6	58	F	S	HM (2.3)	14	+	+	+	PPV*2, SO, Lens extraction	PC-IOL	416	270° retinectomy + SO removal + IVIA	360° pRD	-	30	0.1 (1.0)	WNL	IVIA*2, IVTA*2, mild CO, recurrent VH
7	52	F	R	LP(+)	5	+	+	+	SB, PPV for ERM	PC-IOL	312	180° retinectomy + SO + IVIA	180° pRD	-	10	HM (2.3)	8	
8	74	M	R	LP(+)	45	+	-	+	PPV*2	AC-IOL	312	150° retinectomy + IVIA + C3F8	150° pRD	-	24	CF/10 cm (2.0)	WNL	CO, DSAEK + IOL
9	31	M	R	0.1 (1.0)	18	-	-	-	PPV*3, SB, SO	Aphakia	40	210° retinectomy + ERM, ILM peeling + SO	210° pRD, ERM, MH	-	48	CF/30 cm (1.9)	WNL	SO removal

* Number of surgery; Ant., anterior; CF, counting fingers; CO, corneal opacity; DSAEK, Descemet’s stripping endothelial keratoplasty; ERM, epiretinal membrane; F/U, follow-up; HM, hand motion; ILM, inner limiting membrane; IOP, intraocular pressure; IVIA, intravitreal injection of Avastin; IVTA, intravitreal injection of triamcinolone; LP, light perception; MH, macular hole; NVI, neovascularization of iris; OP, operation; PI, peripheral iridectomy; PPL, pars plana lensectomy; PPV, pars plana vitrectomy; pRD, peripheral rhegmatogenous retinal detachment; SB, scleral buckle; SO, silicone oil; VA, visual acuity; VH, vitreous hemorrhage; WNL, within normal limits.

**Table 2:** Summary of patients’ demographic data.

Characteristics	*n* = 9
Age, mean (range)	52.3 (31–74)
Sex (M/F)	6/3
Pre-Op numbers, mean (range)	2.3 (2–3)
Onset time, mean (range), (weeks)	130.4 (12–416)
IOP (≤5 mmHg/>30 mmHg), (*n*)	2/2
BCVA (LS/HM/CF/>0.01), (*n*)	3/1/2/3
Lens status (Cataract/Pseudophakia/Aphakia), (*n*)	1/5/3
F/U, mean (range), (months)	19.6 (6–48)
Iris rubeosis, (*n*)	7
Hyphema, (*n*)	5
Vitreous hemorrhage, (*n*)	5
Fundus change (pRD > 180°), (*n*)	8
OP procedures (Retinectomy/SO/IVIA), (*n*)	9/7/3
Complications	Corneal decompensation: 3; DSAEK: 1
Final BCVA (LP/HM/CF/> 0.01), (*n*)	0/3/2/4
Final IOP (≤5 mmHg/>30 mmHg), (*n*)	2/0

BCVA, beset-corrected visual acuity; CF, counting fingers; DSAEK, Descemet’s stripping endothelial keratoplasty; F/U, follow-up; HM, hand motion; IOP, intraocular pressure; IVIA, intravitreal injection of Avastin; LP, light perception; OP, operation; pRD, peripheral rhegmatogenous retinal detachment; SO, silicone oil.

## Data Availability

Data are available on reasonable request from the corresponding author on reasonable request. Email: chungmay@ntu.edu.tw.
